# Federated Self-Supervised Few-Shot Face Recognition

**DOI:** 10.3390/jimaging11100370

**Published:** 2025-10-18

**Authors:** Nursultan Makhanov, Beibut Amirgaliyev, Talgat Islamgozhayev, Didar Yedilkhan

**Affiliations:** Smart City Research Center, Astana IT University, Astana 010000, Kazakhstan; n.mahanov@astanait.edu.kz (N.M.); talgat.islamgozhayev@astanait.edu.kz (T.I.); d.yedilkhan@astanait.edu.kz (D.Y.)

**Keywords:** face recognition, federated learning, self-supervised learning, few-shot learning, privacy-preserving machine learning

## Abstract

This paper presents a systematic framework that combines federated learning, self-supervised learning, and few-shot learning paradigms for privacy-preserving face recognition. We use the large-scale CASIA-WebFace dataset for self-supervised pre-training using SimCLR in a federated setting, followed by federated few-shot fine-tuning on the LFW dataset using prototypical networks. Through comprehensive evaluation across six state-of-the-art architectures (ResNet, DenseNet, MobileViT, ViT-Small, CvT, and CoAtNet), we demonstrate that while our federated approach successfully preserves data privacy, it comes with significant performance trade-offs. Our results show 12–30% accuracy degradation compared to centralized methods, representing the substantial cost of privacy preservation. We find that traditional CNNs show superior robustness to federated constraints compared to transformer-based architectures, and that five-shot configurations provide an optimal balance between data efficiency and performance. This work provides important empirical insights and establishes benchmarks for federated few-shot face recognition, quantifying the privacy–utility trade-offs that practitioners must consider when deploying such systems in real-world applications.

## 1. Introduction

Face recognition has emerged as one of the most prominent applications of computer vision and biometric identification systems, experiencing a remarkable evolution from traditional handcrafted feature-based methods to sophisticated deep learning architectures [1,2]. The field has witnessed unprecedented progress with the advent of deep convolutional neural networks (CNNs), particularly ResNet [3]; DenseNet [4]; and, more recently, Vision Transformers [5,6], which have significantly improved recognition accuracy across diverse datasets and challenging real-world scenarios [7,8].

Despite these remarkable achievements, contemporary face recognition systems face several critical challenges that limit their practical deployment and widespread adoption. First, the centralized nature of traditional deep learning approaches requires the collection and aggregation of massive amounts of sensitive biometric data, raising substantial privacy concerns and regulatory compliance issues, particularly in light of stringent data protection regulations such as GDPR and CCPA [9,10]. Second, the computational and communication overhead associated with the training of large-scale models on centralized servers creates bottlenecks in distributed environments where data cannot be easily transferred due to bandwidth limitations or security constraints [11]. Third, the dependency on extensive manually annotated datasets for supervised learning poses significant challenges in terms of annotation costs, time requirements, and scalability to new domains or identities [12].

To address these fundamental limitations, three complementary learning paradigms have emerged as promising solutions. Federated Learning (FL) enables collaborative model training across distributed clients without requiring centralized data collection, thereby preserving data privacy while leveraging the collective knowledge of multiple participants [9,11]. Self-Supervised Learning (SSL) has demonstrated a remarkable capability in learning robust visual representations from unlabeled data by exploiting inherent data structures and relationships, reducing the dependency on manual annotations [13,14,15]. Few-Shot Learning (FSL) addresses the challenge of limited labeled data by allowing models to generalize to new classes or identities with minimal training examples, making it particularly suitable for scenarios where obtaining extensive labeled datasets is impractical [16,17].

The integration of these three paradigms presents a compelling opportunity to create a unified framework that simultaneously addresses privacy preservation, annotation efficiency, and communication optimization in face recognition systems. While federated learning ensures data privacy and enables distributed training, self-supervised learning reduces the reliance on labeled data during the pre-training phase, and few-shot learning enables efficient adaptation to new identities with minimal supervision. This synergistic combination is particularly relevant for real-world applications such as surveillance systems, access control, and mobile authentication, where data privacy, computational efficiency, and rapid deployment are paramount concerns.

Despite the individual success of federated learning [10,11], self-supervised learning [14,15,18], and few-shot learning [19,20,21] in face recognition, their integrated potential remains largely unexplored in the literature. Previous work has primarily focused on addressing these challenges in isolation, failing to take advantage of the complementary strengths of these paradigms. For example, while Kim et al. [11] proposed federated learning for face recognition via self-supervised intrasubject learning, their approach did not incorporate few-shot learning capabilities for efficient adaptation to new identities. Similarly, existing few-shot face recognition methods [20,22] typically assume centralized training scenarios and do not address concerns about privacy preservation or communication efficiency inherent in federated environments.

The integration of these three paradigms introduces several technical challenges that require careful consideration. First, the heterogeneity of data distributions across federated clients can significantly impact the effectiveness of self-supervised pre-training, as the learned representations may not generalize well across different client populations. Second, the communication overhead in federated settings becomes more pronounced when dealing with large-scale self-supervised models, necessitating efficient compression and aggregation strategies. Third, the few-shot learning component must be designed to work effectively with representations learned through federated self-supervised pre-training, which may exhibit different characteristics compared to centrally trained models.

To address these challenges, this paper proposes a novel unified framework that seamlessly integrates federated learning, self-supervised learning, and few-shot learning for privacy-preserving face recognition. Our approach consists of two main phases: (1) a self-supervised pre-training phase using the large-scale CASIA-WebFace dataset [23] to learn robust face representations without manual annotations and (2) a federated few-shot learning phase that efficiently adapts the pre-trained model to downstream face recognition tasks on the LFW dataset [24] with minimal labeled examples while maintaining privacy constraints.

We develop a systematic framework that combines three established paradigms as federated learning, self-supervised learning, and few-shot learning for face recognition. While the integration is primarily sequential rather than deeply synergistic, our comprehensive evaluation across multiple architectures provides valuable insights into the practical deployment of such systems. We acknowledge that this represents an engineering contribution rather than a fundamental algorithmic breakthrough, as we use standard SimCLR for self-supervised pre-training, standard FedAvg for federated learning, and standard prototypical networks for few-shot learning without deep algorithmic innovations.

Through extensive experimental evaluation across six state-of-the-art architectures, we demonstrate that while our federated approach preserves data privacy, it comes with significant performance trade-offs. Our results show 12–30% accuracy degradation compared to centralized methods, representing the substantial cost of privacy preservation. This significant performance degradation raises important questions about the practical viability of such approaches and represents a fundamental limitation that practitioners must carefully consider when deploying federated face recognition systems.

The main contributions of this work can be summarized as follows:

- Comprehensive empirical analysis: We provide the first systematic evaluation of the combination of federated learning, self-supervised learning, and few-shot learning for face recognition across six state-of-the-art architectures, establishing benchmarks for future research.
- Architectural suitability insights: We demonstrate that traditional CNNs (ResNet and DenseNet) show superior robustness to federated constraints compared to transformer-based architectures, with implications for practical deployment decisions.
- Privacy–utility trade-off quantification: We establish benchmarks showing 12–30% accuracy degradation as the cost of privacy preservation, providing guidance for practical deployment decisions and highlighting the substantial limitations of current federated approaches.
- Engineering framework: We develop a working system that successfully integrates three complementary paradigms, serving as a foundation for future algorithmic innovations in federated few-shot learning, while acknowledging the primarily sequential nature of this integration.

The remainder of this paper is organized as follows. Section 2 reviews related work on federated learning, self-supervised learning, and a few-shot learning method for face recognition. Section 3 formally defines the problem and establishes the mathematical framework. Section 4 presents our proposed unified approach with detailed algorithmic descriptions. Section 5 describes the experimental setup and datasets used for evaluation. Section 6 presents comprehensive experimental results and analysis. Finally, Section 7 concludes the paper and discusses future research directions.

## 2. Related Works

### 2.1. Face Recognition

Face recognition has evolved significantly from traditional methods to deep learning approaches. Early techniques, such as geometric feature analysis and texture-based methods (e.g., local binary patterns), relied on manually engineered features for tasks such as detection, extraction, and verification [1,2]. These methods struggled with environmental variations such as lighting changes and occlusions. The advent of deep learning, particularly convolutional neural networks (CNNs), revolutionized the field by automating feature extraction, achieving state-of-the-art accuracy through models such as VGG-Face, FaceNet, and ArcFace [7,8,12]. Hybrid approaches that combine classical and deep learning methods further improved robustness.

Recent advancements focus on addressing scalability and environmental challenges. Federated learning (FL) frameworks such as FedFS enable decentralized training of personalized face recognition models without centralized data aggregation, enhancing privacy through intra-subject self-supervised learning [9,11]. Techniques utilizing cosine similarity and adaptive soft labels have improved the robustness to pose variations and occlusions [12]. In addition, generative adversarial networks (GANs) are being used to synthesize training data in FL settings, mitigating data scarcity while preserving privacy [10].

Limitations persist in three key areas:

- Federated Learning: Communication overhead and heterogeneity across edge devices hinder model convergence, while secure aggregation methods remain computationally intensive.
- Few-Shot Learning: Deep learning models require large datasets, limiting their effectiveness in scenarios with minimal training samples.
- Self-Supervised Learning: While frameworks like FedFS reduce dependency on labeled data, integrating self-supervised objectives with FL introduces complexity in maintaining consistent representation spaces across clients. Furthermore, ethical concerns around bias in training data and high computational demands underscore the need for lightweight and fairer architectures.

### 2.2. Self-Supervised Learning

Self-supervised learning (SSL) has emerged as a transformative paradigm for the learning of robust representations without manual annotations, leveraging intrinsic data structures to generate supervisory signals. Early SSL approaches focused on pretext tasks like image rotation prediction [15,25] and contrastive learning frameworks that maximize similarity between augmented views of data while repelling dissimilar pairs [26,27]. Cross-modal methods, such as cyclic translation and joint image–text pre-training, further expanded SSL’s scope by aligning representations across modalities. These techniques reduce reliance on labeled datasets, making SSL particularly valuable in domains with a scarcity of annotations.

Recent advances in SSL emphasize multimodal and domain-specific innovations. Contrastive frameworks like VG-SSL integrate geo-related image pairs (GeoPair) to enhance visual geo-localization accuracy, achieving performance comparable to that of supervised methods [27]. In medical imaging, SSL optimizes metrics such as the area under the curve (AUC) to balance sensitivity and specificity, which are critical for diagnostic reliability [13]. Multimodal SSL models such as those that combine TimeSformer visual backbones with language models demonstrate superior video captioning performance when trained jointly on images and videos [28]. These advancements highlight SSL’s adaptability to diverse data types and tasks.

Face recognition applications include domain adaptation and keypoint detection. SSL mitigates domain changes by aligning source- and target-domain embeddings through maximization of similarity, improving recognition accuracy under dataset biases [14]. For facial landmark detection in thermal imaging, SSL pretraining with rotation prediction and keypoint regression tasks achieves high precision despite limited labeled data [15]. Semi-supervised frameworks such as self-training LDA integrate affinity propagation to iteratively refine face subspaces using unlabeled samples, outperforming traditional supervised methods in low-data regimes. These applications underscore SSL’s ability to address data scarcity and generalization challenges in face recognition systems [18,29].

### 2.3. Few-Shot Learning

Few-shot learning (FSL) addresses data scarcity by leveraging prior knowledge to rapidly adapt models to new tasks with minimal labeled examples. Traditional approaches relied on hand-crafted features and shallow architectures, but deep learning frameworks now dominate through metric-based, optimization-based, and model-based meta-learning paradigms [16,17].

Meta-learning approaches focus on task generalization through episodic training.

Metric-based methods such as prototypical networks [19] classify samples by computing distances to class prototypes in learned embedding spaces, while matching networks [30] employ attention mechanisms for similarity scoring.

Optimization-based methods (e.g., MAML and Reptile [30]) learn model initialization parameters that enable fast adaptation to new tasks via gradient updates. The Reptile method shows superior accuracy (F1: 0.92) compared to MAML (F1: 0.87) in face recognition tasks [30].

Model-based methods use memory-augmented networks to store and retrieve task-specific information dynamically, enhancing adaptability [17,31].

Applications to face recognition demonstrate FSL’s viability in low-data regimes.

Siamese Networks with contrastive loss achieve 90% accuracy on five-way one-shot tasks by learning discriminative embeddings from single examples [20,21]. Hybrid frameworks combining deep features (e.g., Dlib) with transfer learning reduce dependency on labeled data, achieving 84% accuracy in 50-way face verification [22]. MetaFake [32] detects unseen face forgery techniques with 89.3% accuracy using task-specific prototypes and local feature aggregation, outperforming conventional supervised methods by 12%. Challenges persist in handling intra-class variation and scaling to large identity sets. Although methods like squeeze-and-excitation networks [21] enhance feature sensitivity, computational costs for secure aggregation in federated FSL remain prohibitive.

### 2.4. Combined Approaches

Combined approaches to face recognition seek to take advantage of the strengths of different paradigms to overcome individual limitations, enhancing robustness, accuracy, and applicability in diverse scenarios. These strategies often integrate traditional methods with deep learning; fuse multiple feature extraction techniques; or blend different learning paradigms like federated, few-shot, and self-supervised learning. For example, early methods combined geometric and texture-based features to improve recognition accuracy under varying lighting conditions. More recently, hybrid approaches utilizing deep neural networks for feature extraction combined with machine learning classifiers such as support vector machines (SVMs) have shown promise [33].

In the context of federated learning with self-supervised objectives, authors in [34] proposed *Calibre*, a personalized federated learning framework designed to achieve both fairness and accuracy across clients. *Calibre* method introduces a contrastive prototype adaptation mechanism that calibrates SSL representations by maintaining a balance between generic and client-specific information. The framework addresses the challenge of fuzzy class boundaries in SSL representations under non-IID data distributions through client-specific prototype regularizers and an aggregation algorithm guided by prototypes across clients. Theoretical analysis based on information theory formalizes the generality–personalization tradeoff, providing foundations for design decisions. Extensive experiments on the CIFAR-10, CIFAR-100, and STL-10 datasets demonstrate that *Calibre* achieves state-of-the-art performance in both mean accuracy and fairness across various non-IID settings.

Current integrated approaches focus on feature fusion, network optimization, and multi-modal data integration. Feature fusion combines hand-crafted features (e.g., LBP and HOG) with deep-learned features to capture complementary information, often improving performance compared to using either feature set alone [33,35]. However, these approaches typically assume centralized data availability and do not address the challenges of decentralized learning environments. For network optimization, Binary Particle Swarm Optimization (BPSO) can refine neural network hidden layers to extract relevant features, improving performance on datasets like FERET and LFW [36]. While *Calibre* demonstrates effective integration of SSL and FL, it does not explicitly address the few-shot learning scenario, which is critical for face recognition with limited labeled data per identity.

Limitations of current integrated approaches include the following:

- Computational Complexity: The combination of multiple features and the training of deep networks increases computational demands, limiting real-time applications and deployment on resource-constrained devices.
- Data Dependency: Although some methods reduce the dependency on labeled data, performance still depends on the quality and quantity of training data, especially in federated and few-shot learning scenarios.
- Generalization Issues: Models trained on specific datasets may struggle to generalize to new environments or demographics, necessitating domain adaptation techniques or continuous learning strategies.

Our work addresses these limitations by introducing a framework that integrates federated learning, few-shot learning, and self-supervised learning to improve face recognition in privacy-sensitive and data-scarce environments. Building upon approaches like *Calibre*, we extend the paradigm of prototype-based learning to the federated few-shot setting, specifically addressing the unique challenges of face recognition, including identity preservation across clients, the handling of facial variations in unconstrained environments, and constructing meaningful few-shot episodes from disjoint identity distributions. By leveraging intra-subject self-supervision within a federated setting, we aim to reduce the reliance on labeled data while preserving user privacy. Furthermore, we address the challenge of heterogeneity across edge devices by incorporating lightweight architectures and efficient communication protocols. This approach positions our work at the intersection of multiple cutting-edge paradigms, offering a practical and scalable solution for real-world face recognition applications.

## 3. Problem Formulation

In this section, we formally define the federated face recognition problem and establish the mathematical framework for our approach. We consider a scenario where multiple clients possess private face datasets and aim to collaboratively train a global face recognition model without sharing their raw data.

### 3.1. Notations and Definitions

Let $C=\{C_{1},C_{2},\ldots ,C_{N}\}$ denote a set of *N* clients participating in the federated learning system and *S* represent the central server. Each client ($C_{i}$) has a local dataset ($D_{i}={\left\{\left(x_{i,j},y_{i,j}\right)\right\}}_{j=1}^{|D_{i}|}$, where $x_{i,j}\in R^{H\times W\times 3}$ represents a face image and $y_{i,j}\in Y_{i}$ is the corresponding identity label). Let $D_{\mathrm{global}}={⋃}_{i=1}^{N}D_{i}$ denote the global dataset composed of all client datasets, with a total size of $|D_{\mathrm{global}}|=\sum_{i=1}^{N}\left|D_{i}\right|$. Each client ($C_{i}$) maintains its local dataset ($D_{i}$) with $|D_{i}|$ samples.

We define the global model parameters as $\theta \in R^{d}$, where *d* is the parameter dimension. The face recognition model consists of a feature extractor ($f_{\theta }:R^{H\times W\times 3}\to R^{d_{f}}$) that maps input images to $d_{f}$-dimensional feature representations and a classifier head ($g_{\phi }:R^{d_{f}}\to R^{\left|Y\right|}$) for identity classification, where |Y| is the total number of identities across all clients.

For the few-shot learning setting, we define a support set ($S={\left\{\left(x_{s}^{\left(n\right)},n\right)\right\}}_{n=1}^{N}$) containing *K* labeled examples per class for *N* classes and a query set ($Q={\left\{\left(x_{q}^{\left(m\right)},y_{q}^{\left(m\right)}\right)\right\}}_{m=1}^{M}$) containing *M* test examples. The few-shot learning task is formulated as an *N*-way *K*-shot classification problem.

Federated Learning Problem: Given *N* clients with private datasets (${\left\{D_{i}\right\}}_{i=1}^{N}$), the objective is to learn a global model ($\theta^{*}$) that minimizes federated loss:

(1) $$\theta^{*}=\arg\min_{\theta }L_{fed}\left(\theta \right)=\sum_{i=1}^{N}\frac{|D_{i}|}{\left|D\right|}L_{i}\left(\theta \right)$$

where $L_{i}\left(\theta \right)=\frac{1}{|D_{i}|}\sum_{\left(x,y\right)\in D_{i}}\ell \left(\theta ;x,y\right)$ is the local loss function in the client (*i*) and ℓ(θ;x,y) is the loss for a single data point.

Self-Supervised Learning Objective: For the pre-training phase, we employ contrastive learning to learn meaningful face representations without labels. Given an input image (*x*), we generate two augmented views ($x_{1}=t_{1}\left(x\right)$ and $x_{2}=t_{2}\left(x\right)$) using transformations ($t_{1},t_{2}∼T$). The contrastive loss for a positive pair (i,j) is expressed as follows:

(2) $$L_{ssl}\left(i,j\right)=-\log\frac{\exp(\mathrm{sim}\left(z_{i},z_{j}\right)/\tau )}{\sum_{k=1}^{2B}1\left(k\neq i\right)\exp\left(\mathrm{sim}\left(z_{i},z_{k}\right)/\tau \right)}$$

where $z_{i}=g\left(f_{\theta }\left(x_{i}\right)\right)$ is the projected representation, $\mathrm{sim}\left(u,v\right)=u^{T}v/\left(∥u∥∥v∥\right)$ is the cosine similarity, τ is the temperature parameter, and *B* is the batch size.

Few-Shot Learning Task: Given a support set (S) and a query set (Q), the objective of learning of a few shots is to minimize the classification error on the query set. Using prototypical networks, the class prototype for class *n* is computed as follows: $c_{n}=\frac{1}{|S_{n}|}\sum_{\left(x_{i},y_{i}\right)\in S_{n}}f_{\theta }\left(x_{i}\right)$, where $S_{n}=\left\{\left(x_{i},y_{i}\right)\in S|y_{i}=n\right\}$ is the support set for class *n*. The classification probability for a query sample ($x_{q}$) is expressed as follows:

(3) $$p\left(y=n|x_{q}\right)=\frac{\exp(-d\left(f_{\theta }\left(x_{q}\right),c_{n}\right))}{\sum_{n^{\prime }=1}^{N}\exp\left(-d\left(f_{\theta }\left(x_{q}\right),c_{n^{\prime }}\right)\right)}$$

where d(·,·) is the Euclidean distance function.

### 3.2. System Model

Our federated face recognition system operates under the following architectural framework and constraints:

The system follows a star topology, where the central server (*S*) coordinates the training process among *N* distributed clients. Each client ($C_{i}$) maintains its local dataset ($D_{i}$) and computational resources for local model training. The server aggregates model updates from selected clients and broadcasts the updated global model.

Training Protocol: The training process consists of three phases:

1. Self-Supervised Pre-training: We pre-train a feature extractor using unlabeled face images through contrastive learning.
2. Federated Few-Shot Fine-tuning: The pre-trained model is fine-tuned using limited labeled samples in a federated few-shot learning framework.
3. Evaluation: The final model is evaluated on the following face recognition tasks.

Communication Model: In each communication round (*t*), the server selects a subset of clients ($S_{t}\subseteq C$) based on availability and resource constraints. Selected clients download the current global model parameters ($\theta^{t}$), perform local training for *E* epochs, and upload their model updates ($\Delta \theta_{i}^{t}=\theta_{i}^{t+1}-\theta^{t}$) to the server. The server then aggregates these updates using weighted averaging:

(4) $$\theta^{t+1}=\theta^{t}+\sum_{i\in S_{t}}\frac{|D_{i}|}{\sum_{j\in S_{t}}\left|D_{j}\right|}\Delta \theta_{i}^{t}.$$

The system enforces strict privacy preservation requirements.

- Data Locality: Raw face images never leave the client devices, ensuring that $D_{i}$ remains private to the client ($C_{i}$).
- Model Privacy: Only model parameters or gradients are shared, not intermediate activations or embeddings that could leak sensitive information.

The system addresses multiple forms of heterogeneity:

- Statistical Heterogeneity: Clients may have non-IID data distributions with varying identity compositions and demographic characteristics.
- Temporal Heterogeneity: Clients may join or leave the system dynamically, and their availability patterns can vary.

Under standard assumptions of bounded gradients and Lipschitz continuity, the federated learning algorithm converges to a stationary point of the global objective function. The convergence rate depends on the degree of data heterogeneity, communication frequency, and local update steps.

This problem formulation establishes the theoretical foundation for our federated self-supervised few-shot face recognition approach, addressing the key challenges of privacy preservation, data heterogeneity, and limited labeled data in distributed face recognition systems.

## 4. Methodology

**Figure 1:**
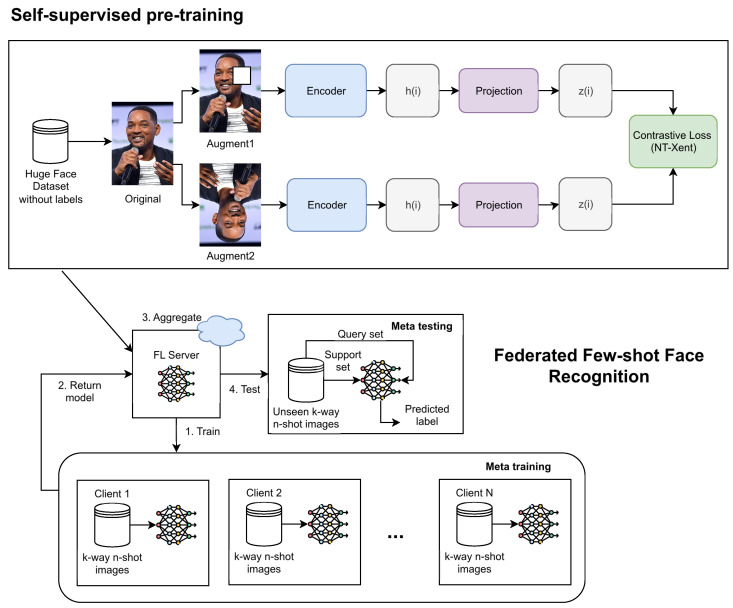
General architecture of federated self-supervised few-shot face recognition.

In this section, we present the detailed methodology of our federated face recognition system in Figure 1, which integrates self-supervised learning and few-shot learning techniques. The approach is designed to address the challenges of privacy preservation, data heterogeneity, and limited labeled data in face recognition tasks.

### 4.1. Federated Learning

We implement Federated Averaging (FedAvg) as our core training algorithm, which enables collaborative model training while keeping face images private on local devices. In FedAvg, a central server coordinates the training process across *K* clients without accessing their local data. Formally, in each round (*t*), the server selects a subset of clients ($S_{t}\subseteq \left\{1,2,\ldots ,K\right\}$) and distributes the global model parameters ($w^{t}$) to them. Each selected client (*k*) then performs local training using their private face dataset ($D_{k}$) to minimize the loss function:  

(5) $$\min_{w}L_{k}\left(w\right)=\frac{1}{|D_{k}|}\sum_{\left(x,y\right)\in D_{k}}\ell \left(w;x,y\right)$$

where ℓ(w;x,y) is the loss for a single data point. After completing *E* local epochs, each client sends their updated model parameters ($w_{k}^{t+1}$) back to the server, which aggregates them using a weighted average:

(6) $$w^{t+1}=\sum_{k\in S_{t}}\frac{|D_{k}|}{\sum_{j\in S_{t}}\left|D_{j}\right|}w_{k}^{t+1}.$$

This approach enables our face recognition system to learn from diverse, distributed facial data while preserving privacy and addressing the heterogeneity challenges inherent in face datasets across different clients.

### 4.2. Self-Supervised Pre-Training

In our federated face recognition system, we adopt SimCLR (Simple Framework for Contrastive Learning of Visual Representations) as our self-supervised pre-training algorithm. SimCLR learns representations by maximizing agreement between differently augmented views of the same data example via a contrastive loss in the latent space. For a batch of *N* face images, we generate 2N augmented examples by applying random transformations, i.e., t∼T (such as cropping, flipping, and color distortion), to each image. Each augmented example is then encoded through a base encoder (f(·)) and a projection head (g(·)) to obtain the representation ($z_{i}=g\left(f\left(x_{i}\right)\right)$). The contrastive loss for a positive pair (i,j) is defined as follows:

(7) $$L_{i,j}=-\log\frac{\exp(\mathrm{sim}\left(z_{i},z_{j}\right)/\tau )}{\sum_{k=1}^{2N}1\left(k\neq i\right)\exp\left(\mathrm{sim}\left(z_{i},z_{k}\right)/\tau \right)}$$

where $\mathrm{sim}\left(u,v\right)=u^{T}v/∥u∥∥v∥$ denotes the cosine similarity between two vectors and τ is a temperature parameter. In the federated setting, each client computes this loss locally based on their private data, and only model updates are shared during aggregation, preserving data privacy while enabling collaborative learning of facial representations.

### 4.3. Few-Shot Learning

Our few-shot learning approach utilizes prototypical networks, which learn a metric space where classification can be performed by computing distances to prototype representations of each class. In the N-way, K-shot setting, each class prototype ($c_{n}$) is computed as the mean embedding of its support examples:

(8) $$c_{n}=\frac{1}{K}\sum_{\left(x_{i},y_{i}\right)\in S_{n}}f_{\theta }\left(x_{i}\right)$$

where $S_{n}$ is the support set for class *n* and $f_{\theta }$ is the embedding function. Given a query image ($x_{q}$), classification is performed by calculating the probability distribution over classes based on softmax-normalized distances:

(9) $$p_{\theta }\left(y=n|x_{q}\right)=\frac{\exp(-d\left(f_{\theta }\left(x_{q}\right),c_{n}\right))}{\sum_{n^{\prime }=1}^{N}\exp\left(-d\left(f_{\theta }\left(x_{q}\right),c_{n^{\prime }}\right)\right)}$$

where d(·,·) is a distance function—typically, a Euclidean distance. This approach is particularly effective for face recognition with limited samples per identity, as it leverages the distance-based decision boundaries that align well with the embedding-space structure of facial features.

### 4.4. Federated Few-Shot Fine-Tuning

In our federated few-shot learning framework, we extend the prototypical network approach to the federated setting. After pre-training the model using self-supervised learning, we implement a fine-tuning protocol where clients collaborate to improve few-shot recognition performance. Formally, in each round (*t*) of federated fine-tuning, the server selects a subset of clients ($S_{t}\subseteq \left\{1,2,\ldots ,K\right\}$) according to the selection probability ($p_{k}$) for each client (*k*). Each selected client performs few-shot learning on their local data, computing class prototypes for their $N_{k}$ local classes:

(10) $$c_{k,n}^{t}=\frac{1}{|S_{k,n}|}\sum_{\left(x_{i},y_{i}\right)\in S_{k,n}}f_{\theta^{t}}\left(x_{i}\right)$$

where $S_{k,n}$ is the support set for class *n* in client *k* and $f_{\theta^{t}}$ is the embedding function with parameters ($\theta^{t}$) from the global model. Each client then computes the loss based on their query set ($Q_{k}$) using these prototypes:

(11) $$L_{k}\left(\theta^{t}\right)=\frac{1}{|Q_{k}|}\sum_{\left(x_{q},y_{q}\right)\in Q_{k}}-\log\frac{\exp(-d\left(f_{\theta^{t}}\left(x_{q}\right),c_{k,y_{q}}^{t}\right))}{\sum_{n=1}^{N_{k}}\exp\left(-d\left(f_{\theta^{t}}\left(x_{q}\right),c_{k,n}^{t}\right)\right)}.$$

The client updates its local model parameters via gradient descent:

(12) $$\theta_{k}^{t+1}=\theta^{t}-\eta \nabla L_{k}\left(\theta^{t}\right)$$

where η is the learning rate. The server then aggregates these updates using a weighted average:

(13) $$\theta^{t+1}=\sum_{k\in S_{t}}\frac{|Q_{k}|}{\sum_{j\in S_{t}}\left|Q_{j}\right|}\theta_{k}^{t+1}.$$

This iterative process continues until convergence, which is defined as occurring when the change in global model parameters falls below a threshold (ϵ: $∥\theta^{t+1}-\theta^{t}∥<\epsilon$). This process is detailed in Algorithm 1.
**Algorithm 1** Federated Few-Shot Fine-tuning  1:**Input:** Pre-trained model parameters $\theta^{0}$, number of clients *K*, number of rounds *T*, local learning rate η, client selection probability ${\left\{p_{k}\right\}}_{k=1}^{K}$  2:**Output:** Fine-tuned global model parameters $\theta^{T}$  3:**for** each round t=0,1,…,T−1 **do**  4:    Server selects subset of clients $S_{t}$ according to probabilities $\left\{p_{k}\right\}$  5:    Server sends global model parameters $\theta^{t}$ to selected clients  6:    **for** each client $k\in S_{t}$ in parallel **do**  7:        **for** each local class $n=1,2,\ldots ,N_{k}$ **do**  8:             Compute prototype $c_{k,n}^{t}=\frac{1}{|S_{k,n}|}\sum_{\left(x_{i},y_{i}\right)\in S_{k,n}}f_{\theta^{t}}\left(x_{i}\right)$  9:        **end for**10:        Compute loss $L_{k}\left(\theta^{t}\right)$ on query set $Q_{k}$ using prototypes11:        Update local model: $\theta_{k}^{t+1}=\theta^{t}-\eta \nabla L_{k}\left(\theta^{t}\right)$12:        Send updated model parameters $\theta_{k}^{t+1}$ to server13:    **end for**14:    Server aggregates updates: $\theta^{t+1}=\sum_{k\in S_{t}}\frac{|Q_{k}|}{\sum_{j\in S_{t}}\left|Q_{j}\right|}\theta_{k}^{t+1}$15:    **if** $∥\theta^{t+1}-\theta^{t}∥<\epsilon$ **then**16:        break                  ▹ Convergence achieved17:    **end if**18:**end for**19:**return**$\theta^{T}$

## 5. Experimental Setup

This section describes the experimental configuration used to evaluate our federated self-supervised few-shot face recognition approach. We detail the datasets, implementation specifics, baseline comparisons, and evaluation protocols.

### 5.1. Datasets

We utilize the CASIA-WebFace dataset [23] for self-supervised pre-training, which contains 494,414 face images of 10,575 identities collected from the Web. The dataset provides diverse facial variations in terms of pose, illumination, expression, and background, making it suitable for the learning of robust face representations. For federated learning simulation, we partition the dataset across 20 clients according to a Dirichlet distribution with a concentration parameter of α=0.1 to simulate non-IID data heterogeneity. Each client is assigned a disjoint subset of identities, ensuring no identity overlap across clients.

After self-supervised pre-training, the global encoder obtained from the federated learning stage is used for federated few-shot fine-tuning and evaluation on the Labeled Faces in the Wild (LFW) dataset [24]. LFW contains 13,233 face images of 5749 identities collected from news articles on the Internet. The dataset is particularly challenging due to unconstrained conditions, including variations in lighting, pose, expression, and image quality. For the federated few-shot evaluation, we similarly employ 20 clients with an IID data distribution to ensure each client has sufficient examples for the construction of meaningful few-shot episodes while maintaining disjoint identities across clients.

Within each client, few-shot episodes are constructed locally following the standard *N*-way *K*-shot protocol. We evaluate 3-way and 5-way classification tasks with 3-shot, 5-shot, and 10-shot settings. For each episode, *N* identities are randomly sampled from the client’s local test set, with *K* images per identity selected for the support set and the remaining images used as queries. We ensure that the support and query sets contain disjoint images of the same identities. Each experimental configuration is evaluated with 100 episodes to ensure statistical significance.

### 5.2. Implementation Details

We evaluate our approach using multiple state-of-the-art architectures—namely, ResNet [3], DenseNet [4], MobileViT [37], ViT-Small [5], CvT [6], and CoAtNet [38]—to demonstrate generalizability.

For each architecture, we conduct experiments with two initialization strategies: (1) using models pre-trained on ImageNet and (2) using models trained from scratch. The feature extractor outputs 512-dimensional embeddings, which are used for both self-supervised pre-training and few-shot fine-tuning.

Our experimental configuration follows these specifications:

- We select 5 of 20 clients randomly for each communication round, with each selected client performing 20 local training epochs.
- We use the Adam optimizer with an initial learning rate of $1\times 10^{-3}$ for self-supervised pre-training and $1\times 10^{-4}$ for few-shot fine-tuning. The learning rate decays by a factor of 0.1 every 50 communication rounds.
- The local batch size is set to 32 for pre-training and 16 for few-shot learning to accommodate memory constraints.
- We conduct 200 rounds for self-supervised pre-training and 20 rounds with 3 local epochs for federated few-shot fine-tuning.
- For contrastive learning, we set τ=0.07 based on empirical validation.

During self-supervised pre-training, we apply the following augmentation techniques to generate positive pairs:

- Random horizontal flipping with a probability of 0.5;
- Random rotation within ±15 degrees;
- Color jittering (brightness: 0.4; contrast: 0.4; saturation: 0.4; hue: 0.1)
- Random grayscale conversion with a probability of 0.2;
- Gaussian blur with a kernel size of 3 and a probability of 0.5;
- Random resized cropping to 224×224 pixels.

For few-shot fine-tuning, we apply minimal augmentation, consisting only of random horizontal flipping and normalization to preserve the integrity of facial features.

Our training follows a three-stage protocol:

1. Self-Supervised Pre-training Stage: We train the feature extractor using SimCLR on unlabeled CASIA-WebFace data for 200 communication rounds.
2. Federated Few-Shot Fine-tuning Stage: The pre-trained model is fine-tuned using prototypical networks on labeled LFW data for 20 communication rounds.
3. Evaluation Stage: The final model is evaluated on a few-shot recognition tasks using the constructed episodes.

### 5.3. Baselines

We compare our federated self-supervised few-shot learning approach with several baseline methods to demonstrate its effectiveness.

Centralized Few-Shot Learning: This baseline represents the upper-bound performance where all data is centrally available. We train prototypical networks on the entire CASIA-WebFace dataset for pre-training, followed by few-shot fine-tuning on LFW. This approach uses the same network architectures and hyperparameters but without federated constraints.

Federated Self-Supervised Few-Shot Learning: This hybrid approach performs centralized self-supervised pre-training on CASIA-WebFace, followed by federated few-shot fine-tuning on LFW. This baseline helps isolate the contribution of federated pre-training. Each client trains independently on their local data without any collaboration. This baseline demonstrates the importance of federated collaboration, especially when local data is limited.

### 5.4. Evaluation Metrics

We evaluated our approach using the following metrics. We report the mean accuracy and the 95% confidence intervals in 100 episodes for each few-shot configuration (3-way/5-way with 3-shot/5-shot/10-shot configurations). This provides a comprehensive evaluation of recognition performance under different difficulty levels. Furthermore, we measure the total communication cost in terms of the transmitted model parameters and the number of communication rounds required to achieve the target performance levels. All experiments are conducted on eight NVIDIA L4 GPUs with 23GB memory, and we obtain the maximum results with fixed seeds to ensure reproducibility.

## 6. Results and Analysis

This section presents a systematic evaluation of our federated self-supervised few-shot face recognition framework. We begin by presenting the overall experimental setup and performance metrics, followed by detailed analyses of architectural comparisons, the impact of federated learning constraints, pre-training strategies, and few-shot learning dynamics. Finally, we discuss practical implications for real-world deployment.

### 6.1. Experimental Setup and Overall Performance

We evaluate six state-of-the-art architectures—ResNet, DenseNet, MobileViT, ViT-Small, CvT, and CoAtNet—under both centralized and federated learning paradigms. Each architecture is tested with and without ImageNet pre-training across 3-way and 5-way classification tasks, using 3-shot, 5-shot, and 10-shot configurations. Table 1 and Table 2 present the comprehensive results for centralized and federated settings, respectively.

The centralized setting (Table 1) consistently outperforms the federated counterpart (Table 2) across all architectures and configurations, as expected, given the availability of complete data and the absence of communication constraints. However, the federated approach demonstrates competitive performance while preserving privacy, with performance gaps varying significantly across architectures and initialization strategies. Among all evaluated architectures, MobileViT with ImageNet pre-training achieves the highest performance in the centralized setting, reaching 99.92% accuracy in the five-way, five-shot configuration. This exceptional performance is attributed to MobileViT’s hybrid architecture, which effectively combines the inductive biases of convolutional layers with the global modeling capabilities of transformers for face recognition tasks. Conversely, ViT-Small exhibits the poorest performance in centralized settings, particularly in three-way tasks (54.20% for 3-shot), indicating that pure transformer architectures require more extensive pre-training or larger model sizes to achieve competitive performance in few-shot scenarios.

### 6.2. Architectural Comparison and Robustness

The performance characteristics of different architectural families reveal important insights into their suitability for federated few-shot face recognition. ResNet and DenseNet demonstrate relatively modest performance degradation when transitioning from centralized to federated settings (approximately 12–15% average drop), indicating their robustness to federated constraints. This resilience is attributed to their well-established architectural designs and proven effectiveness in distributed learning scenarios. Both architectures maintain consistent performance across different shot configurations, with ResNet achieving 85.20% and DenseNet reaching 85.30% on federated three-way, three-shot tasks without pre-training. Their inductive biases, particularly translation equivariance and local connectivity, appear well-suited for face recognition under distributed constraints.

Pure transformer architectures, as exemplified by ViT-Small, struggle significantly in few-shot scenarios. In centralized three-way, three-shot settings, ViT-Small achieves only 54.20% accuracy with pre-training, suggesting that the global attention mechanism may not be optimal when training data are limited. However, ViT-Small shows interesting behavior in federated settings, achieving 91.30% accuracy in 3-way, 10-shot configurations, indicating potential for performance improvement with sufficient support examples. This variability suggests that transformers require careful configuration in few-shot federated contexts.

Hybrid architectures that combine convolutional and attention mechanisms (MobileViT, CvT, and CoAtNet) exhibit diverse trade-offs. MobileViT achieves exceptional performance in centralized settings but experiences substantial degradation in federated scenarios. In particular, MobileViT drops from 85.30% to 78.02% in a five-way, three-shot configuration when trained from scratch, suggesting that lightweight architectures may be more sensitive to data heterogeneity and limited local training data. Despite these challenges, MobileViT with ImageNet pre-training maintains the highest federated performance (91.36% in a 5-way, 10-shot configuration), demonstrating the value of strong initialization. CvT shows strong dependence on pre-training, with dramatic performance differences between initialized and randomly initialized models. The performance drop from 78.64% to 40.26% in the federated five-way, three-shot configuration without pre-training highlights the critical importance of initialization strategies for this architecture in distributed learning. CoAtNet demonstrates more balanced performance across different settings, maintaining relatively stable accuracy without exhibiting extreme sensitivity to either pre-training or federated constraints. This stability suggests that CoAtNet’s architectural design principles are well-suited for diverse learning scenarios.

Contrary to expectations, model complexity does not directly correlate with federated performance degradation. Lightweight models like MobileViT show substantial performance drops, while more complex models like CoAtNet maintain relatively stable performance. This suggests that architectural design principles, rather than parameter count, are more critical to federated learning success.

### 6.3. Impact of Federated Learning Constraints

The transition from centralized to federated learning reveals several fundamental challenges that affect model performance differently across architectures. The federated results reveal several key challenges inherent to distributed learning. First, the non-IID data distribution across clients creates statistical heterogeneity that impedes convergence to optimal solutions. Second, limited communication rounds and local training epochs constrain the model’s ability to fully leverage collective knowledge across clients. Third, the privacy-preserving nature of federated learning prevents direct data sharing, limiting the model’s exposure to diverse facial variations that are crucial for robust face recognition.

Different architectures demonstrate varying degrees of adaptation to federated constraints. MobileViT with ImageNet pre-training maintains relatively high performance in federated settings (91.36% in the 5-way 10-shot, configuration), suggesting that pre-trained representations provide a strong foundation that can be effectively fine-tuned in distributed scenarios. This finding underscores the importance of transfer learning in mitigating federated learning challenges. Conversely, architectures without appropriate initialization struggle significantly. CvT without pre-training shows dramatic performance degradation (from 78.64% to 40.26% in the five-way, three-shot configuration), highlighting the critical importance of initialization strategies in federated learning. This sensitivity suggests that certain architectural designs require stronger inductive biases or better initialization to overcome data heterogeneity in federated settings.

### 6.4. Effect of Pre-Training Strategies

The impact of ImageNet pre-training varies substantially across centralized and federated paradigms, revealing architecture-dependent patterns. In centralized settings, the impact of ImageNet pre-training is architecture-dependent. MobileViT shows the most dramatic improvement with pre-training (from 85.30% to 99.80% in the five-way, three-shot configuration), indicating that lightweight architectures particularly benefit from pre-trained representations. This substantial improvement (14.50 percentage points) demonstrates that pre-trained features effectively compensate for the limited capacity of lightweight models.

Surprisingly, ResNet and DenseNet show a minimal or even negative impact from ImageNet pre-training in some configurations, suggesting that self-supervised pre-training on domain-specific face data (CASIA-WebFace) may be more beneficial than generic ImageNet features for face recognition tasks. For instance, ResNet without pre-training achieves 97.23% accuracy compared to 96.63% with pre-training in the three-way, three-shot configuration, indicating that domain-specific self-supervised learning provides more relevant features for face recognition.

The federated learning paradigm reveals different pre-training dynamics. For most architectures, ImageNet pre-training provides modest improvements or maintains comparable performance. However, CvT shows a stark contrast, where pre-training becomes crucial for reasonable performance (83.76% vs. 40.26% in the five-way, three-shot configuration). This 43.50-percentage-point difference represents the largest pre-training effect observed in our experiments, emphasizing that certain hybrid architectures critically depend on strong initialization to overcome federated learning challenges, particularly under limited data availability for each client.

The mixed results regarding the effectiveness of ImageNet pre-training suggest that domain-specific self-supervised pre-training on face data may be more valuable than generic pre-training for face recognition tasks. This finding supports our approach of using SimCLR on the CASIA-WebFace dataset for pre-training, as it provides face-specific representations that are more aligned with the downstream task requirements.

### 6.5. Few-Shot Learning Dynamics

The analysis of different shot configurations reveals important insights about learning behavior and optimal data efficiency. Generally, increasing the number of shots improves performance across most configurations, but the improvement patterns vary significantly. In centralized settings, most architectures show substantial improvements from 3-shot to 5-shot configurations, with diminishing returns from 5-shot to 10-shot configurations. For example, the accuracy of ResNet with ImageNet pre-training improves from 97.90% to 99.64% (from the three-shot to five-shot configuration) but only marginally to 99.80% (from the 5-shot to 10-shot configuration) in 5-way classification. This saturation pattern suggests that the five-shot configuration may represent an optimal balance between data efficiency and performance for most architectures, with additional support examples providing limited marginal benefit.

Interestingly, some architectures perform better in five-way than three-way tasks, which contradicts the typical expectation that fewer classes should be easier to distinguish. This phenomenon is particularly evident in CoAtNet without pre-training (97.80% in the five-way, three-shot configuration vs. 74.93% in the three-way, three-shot configuration). This 22.87-percentage-point difference may be attributed to the specific characteristics of the LFW dataset and episode construction, where five-way tasks potentially provide more diverse and representative samples that facilitate better prototype formation in the embedding space.

In federated settings, the few-shot learning dynamics become more complex. Performance improvements with an increased number of shots are less consistent, and some configurations show performance degradation with more shots. For instance, ResNet with ImageNet pre-training shows decreasing performance from 5-shot (89.66%) to 10-shot (86.26%) configurations in 3-way tasks. This unexpected degradation suggests that federated learning may suffer from overfitting to local data distributions when provided with more shots, highlighting the need for better regularization strategies in federated few-shot learning. The non-IID nature of client data may cause models to overfit to local biases rather than learning generalizable representations.

### 6.6. Practical Implications and Deployment Guidelines

Based on our comprehensive experimental analysis, we provide actionable recommendations for the deployment of federated face recognition systems in diverse real-world scenarios. For scenarios where communication efficiency is paramount, the ResNet and DenseNet architectures provide the best balance between performance and robustness. These architectures achieve approximately 85% accuracy in federated three-way, three-shot settings while maintaining reasonable parameter counts and stable convergence. When computational resources are limited, MobileViT with careful pre-training strategies may be suitable, despite its federated performance challenges. With proper ImageNet initialization, MobileViT can achieve over 91% accuracy on federated 5-way, 10-shot tasks while offering substantial reductions in communication overhead. For applications requiring the highest possible accuracy and where communication costs are less critical, hybrid architectures like CoAtNet offer promising alternatives. CoAtNet demonstrates consistent performance across diverse settings, making it suitable for scenarios where prediction reliability is more important than communication efficiency.

Our results suggest that domain-specific self-supervised pre-training is more valuable than generic ImageNet pre-training for face recognition tasks. Organizations deploying federated face recognition systems should prioritize collecting diverse unlabeled face data for self-supervised pre-training rather than relying solely on ImageNet initialization. This recommendation is particularly important for the ResNet and DenseNet architectures, which show minimal benefit or negative transfer from ImageNet pre-training while demonstrating strong performance with domain-specific self-supervised learning on the CASIA-WebFace dataset.

The five-shot configuration appears to provide an optimal balance between data efficiency and performance across most architectures and settings. This finding is practically significant, as it suggests that federated face recognition systems can achieve reasonable performance with relatively few labeled examples per identity, reducing the annotation burden on participating clients. Specifically, our results indicate that moving from a 5-shot to 10-shot configuration provides minimal accuracy gains (typically less than 2%) while doubling the annotation requirements, making the five-shot configuration the preferred configuration for cost-effective deployment.

To mitigate the performance degradation observed in federated settings, we recommend (1) implementing robust aggregation schemes that account for data heterogeneity across clients; (2) incorporating regularization techniques to prevent overfitting to local distributions, particularly in higher-shot configurations; and (3) employing domain-specific pre-training to provide strong initialization that reduces sensitivity to federated constraints. These strategies are especially critical for lightweight and hybrid architectures that show higher sensitivity to distributed learning challenges. The different architectures have varying parameter counts, directly affecting communication costs in federated learning, and our observations suggest that convolutional architectures tend to show more stable convergence, whereas transformer-based models may require more communication rounds to achieve comparable performance, with important implications for the total communication cost and the feasibility of deployment.

### 6.7. Comparison with Existing Literature

To provide context for our results, we compare our findings with existing work in federated face recognition and few-shot learning, acknowledging the challenges in direct comparison due to differences in experimental setups, datasets, and evaluation protocols.

Kim et al. [11] reported federated face recognition results using self-supervised intra-subject learning, achieving approximately 85–90% accuracy on LFW in centralized few-shot settings. Our centralized results with traditional CNNs (ResNet and DenseNet) indicate comparable or superior performance (95–99% in few-shot settings), validating our experimental setup. However, their federated results showed less performance degradation (5–10%) compared to our 12–30% drop, suggesting that their intra-subject approach may be more robust to federated constraints than our inter-subject contrastive learning approach.

In the broader few-shot face recognition literature, existing methods report varying performance depending on the dataset and protocol. Chanda et al. [20] achieved 90% accuracy on five-way, one-shot tasks using Siamese networks—comparable to our centralized results but significantly higher than our federated performance (60–85% depending on the architecture). Similarly, Ranadive et al. [22] reported 84% accuracy in 50-way face verification, which presents a more challenging scenario than our 3-way and 5-way tasks, making direct comparison difficult but highlighting the complexity of few-shot face recognition across different experimental settings.

Our approach to self-supervised learning aligns with recent trends in the field. Lin et al. [14] demonstrated the effectiveness of domain-specific pre-training for face recognition, and our finding that domain-specific self-supervised pre-training often outperforms ImageNet pre-training supports their observations. This consistency across different studies validates the importance of task-specific representation learning in face recognition applications.

The substantial performance degradation we observe (12–30%) in federated settings is notably higher than existing federated learning works in other domains, which typically report 5–15% degradation. This disparity suggests that face recognition may be particularly sensitive to the data heterogeneity and communication constraints inherent in federated learning, representing a significant challenge for practical deployment that extends beyond general federated learning limitations.

Our architectural analysis reveals interesting contrasts with current trends in centralized face recognition. While transformer-based models often achieve state-of-the-art results in centralized settings, our findings show that traditional CNNs (ResNet and DenseNet) outperform transformer-based architectures in federated few-shot scenarios. This discrepancy indicates that architectural preferences may differ significantly between centralized and federated learning environments, with implications for system design in distributed settings.

Several factors limit direct comparison with existing literature. Differences in datasets and evaluation protocols make quantitative comparison challenging, while many existing federated face recognition works do not report few-shot performance metrics. Additionally, the specific combination of federated learning, self-supervised learning, and few-shot learning has limited prior work for direct comparison. These limitations underscore the need for standardized benchmarks in federated few-shot face recognition to enable more meaningful comparisons across different approaches and facilitate progress in this emerging research area.

### 6.8. Limitations and Future Directions

Our analysis reveals several critical limitations that fundamentally challenge the practical viability of our approach and point to important future research directions.

The most significant limitation is the substantial 12–30% performance degradation in federated settings, which raises serious questions about practical deployment. This performance gap is not merely a technical challenge to be optimized away but represents a fundamental trade-off inherent in privacy-preserving distributed learning. For applications requiring high accuracy (e.g., security systems and access control), this degradation may render the approach unsuitable for real-world deployment. Future work must honestly address whether federated face recognition can ever achieve the accuracy levels required for critical applications.

Our approach suffers from several methodological shortcomings that limit its contribution. The integration of the three paradigms is primarily sequential rather than synergistic, missing opportunities for deeper algorithmic innovation. We use standard aggregation methods (weighted averaging) without addressing the fundamental challenges of statistical heterogeneity across clients. The lack of novel algorithmic contributions means that the work primarily demonstrates the limitations of existing techniques rather than advancing the state of the art.

The communication overhead and convergence challenges observed in our experiments suggest that the approach may not scale effectively to larger federated networks. The requirement for multiple communication rounds and the sensitivity to client heterogeneity create practical barriers to deployment. Future work should address whether federated few-shot learning can achieve acceptable performance with realistic communication constraints and client availability patterns.

Our evaluation is primarily limited to the LFW dataset following self-supervised pre-training on CASIA-WebFace. While this provides a standardized benchmark for comparison, it constrains our ability to assess model performance across diverse demographic distributions, image quality variations, and environmental conditions. Future work should validate these findings across additional face recognition benchmarks to establish broader generalizability. An important direction for future research involves extending the evaluation to diverse face recognition benchmarks, including AgeDB-30 for age-invariant recognition and CFP-FP for pose robustness. Such multi-dataset validation would provide a more comprehensive understanding of how federated constraints affect model performance across different recognition challenges.

Given these limitations, future research should focus on (1) developing fundamentally new approaches that achieve better privacy–utility trade-offs rather than incremental improvements to existing methods, (2) establishing theoretical frameworks that explain the performance degradation and guide algorithm design, (3) creating more realistic evaluation protocols that capture the complexity of real-world federated environments, and (4) honestly assessing whether federated face recognition can meet the accuracy requirements of practical applications or if alternative privacy-preserving approaches should be pursued.

While this work provides valuable empirical insights, the substantial performance limitations significantly reduce its practical impact. The 12–30% accuracy degradation represents a fundamental barrier to adoption that cannot be easily overcome through incremental improvements. Future work should prioritize developing approaches that can achieve acceptable accuracy levels while preserving privacy or clearly define the limited application domains where such performance trade-offs are acceptable.

## 7. Conclusions

This paper presents a systematic empirical study that combines federated learning, self-supervised learning, and few-shot learning paradigms for privacy-preserving face recognition. While our approach successfully combines three established paradigms, the integration is primarily sequential rather than deeply synergistic.

The most significant finding is the substantial privacy–utility trade-off inherent in federated learning: our results show consistent performance degradation of 12–30% compared to centralized methods, representing the substantial cost of privacy preservation. This significant performance degradation raises important questions about the practical viability of such approaches and represents a fundamental limitation that practitioners must carefully consider. Traditional convolutional networks (ResNet and DenseNet) demonstrate superior robustness to federated constraints, with modest 12–15% performance drops, while transformer-based architectures suffer more severe degradation, with some configurations losing over 30% accuracy.

Our empirical analysis reveals several important findings for practitioners. Domain-specific self-supervised pre-training on facial data often proves more beneficial than ImageNet pre-training, particularly for established architectures, validating the use of SimCLR on face-specific datasets. Additionally, five-shot configurations consistently provide optimal balance between data efficiency and recognition performance across most architectures and settings, suggesting that federated face recognition systems can achieve reasonable performance with relatively few labeled examples per identity.

This research makes valuable empirical contributions by establishing benchmarks for federated few-shot face recognition, quantifying privacy–utility trade-offs, and providing practical insights about architectural suitability for distributed learning scenarios. This work serves as a foundation for future algorithmic innovations in federated few-shot learning while honestly acknowledging the substantial challenges and limitations that remain in making such systems practically viable for real-world deployment.

## Figures and Tables

**Table 1:** Centralized ProtoNet results.

Model	ImgNet Pre-Trained	3-Way			5-Way		
		3-Shot	5-Shot	10-Shot	3-Shot	5-Shot	10-Shot
ResNet	yes	96.63	99.10	99.60	97.90	99.64	99.80
	no	*97.23*	99.33	**99.80**	*98.14*	*99.78*	99.80
DenseNet	yes	96.33	99.37	99.67	96.74	99.60	99.64
	no	95.77	*99.67*	**99.80**	97.42	99.60	**99.88**
MobileViT	yes	**99.40**	**99.80**	*99.73*	**99.80**	**99.92**	*99.84*
	no	86.43	92.63	96.20	85.30	94.16	97.12
ViT small	yes	54.20	71.60	78.27	58.82	83.54	90.64
	no	58.17	68.87	76.73	59.44	83.40	89.36
CvT	yes	62.00	73.27	73.27	87.48	97.78	97.92
	no	38.57	67.77	74.80	78.64	93.88	96.28
CoAtNet	yes	86.90	96.10	96.80	97.68	99.36	99.16
	no	74.93	78.53	84.07	97.80	98.88	94.00

Bold values indicate the highest accuracy achieved across all models for each shot configuration, while underlined with italic values denote the second-highest performance.

**Table 2:** Federated ProtoNet results.

Model	ImgNet Pre-Trained	3-Way			5-Way		
		3-Shot	5-Shot	10-Shot	3-Shot	5-Shot	10-Shot
ResNet	yes	84.50	*89.66*	86.26	81.64	84.88	81.78
	no	*85.20*	87.56	84.30	81.68	85.20	82.76
DenseNet	yes	83.40	89.50	82.40	82.10	86.02	84.72
	no	**85.30**	88.36	82.20	82.30	86.24	85.12
MobileViT	yes	80.30	**92.13**	*90.76*	**89.20**	**91.16**	**91.36**
	no	79.87	82.73	82.70	78.02	80.00	79.50
ViT small	yes	62.66	65.10	**91.30**	82.80	60.72	60.96
	no	79.86	65.73	88.50	*84.58*	59.14	62.68
CvT	yes	78.23	80.36	79.83	83.76	*90.28*	*88.90*
	no	52.80	54.90	58.60	40.26	46.10	52.40
CoAtNet	yes	75.53	84.23	78.40	84.10	81.96	81.64
	no	78.70	81.06	76.90	83.92	84.38	77.82

Bold values indicate the highest accuracy achieved across all models for each shot configuration, while underlined with italic values denote the second-highest performance.

## Data Availability

The data presented in this study are openly available in Kaggle at https://www.kaggle.com/datasets/debarghamitraroy/casia-webface (accessed on 15 October 2025).
